# Evidence for Multiple Diagenetic Episodes in Ancient Fluvial‐Lacustrine Sedimentary Rocks in Gale Crater, Mars

**DOI:** 10.1029/2019JE006295

**Published:** 2020-08-13

**Authors:** C. N. Achilles, E. B. Rampe, R. T. Downs, T. F. Bristow, D. W. Ming, R. V. Morris, D. T. Vaniman, D. F. Blake, A. S. Yen, A. C. McAdam, B. Sutter, C. M. Fedo, S. Gwizd, L. M. Thompson, R. Gellert, S. M. Morrison, A. H. Treiman, J. A. Crisp, T. S. J. Gabriel, S. J. Chipera, R. M. Hazen, P. I. Craig, M. T. Thorpe, D. J. Des Marais, J. P. Grotzinger, V. M. Tu, N. Castle, G. W. Downs, T. S. Peretyazhko, R. C. Walroth, P. Sarrazin, J. M. Morookian

**Affiliations:** ^1^ NASA Goddard Space Flight Center Greenbelt MD USA; ^2^ NASA Johnson Space Center Houston TX USA; ^3^ Department of Geosciences University of Arizona Tucson AZ USA; ^4^ NASA Ames Research Center Moffett Field CA USA; ^5^ Planetary Science Institute Tucson AZ USA; ^6^ Jet Propulsion Laboratory California Institute of Technology Pasadena CA USA; ^7^ Jacobs at NASA Johnson Space Center Houston TX USA; ^8^ Department of Earth and Planetary Sciences University of Tennessee, Knoxville Knoxville TN USA; ^9^ Department of Earth Sciences University of New Brunswick Fredericton New Brunswick Canada; ^10^ Department of Physics University of Guelph Guelph Ontario Canada; ^11^ Carnegie Institute for Science Washington DC USA; ^12^ Lunar and Planetary Institute Houston TX USA; ^13^ School of Earth and Space Exploration Arizona State University Tempe AZ USA; ^14^ Chesapeake Energy Oklahoma City OK USA; ^15^ Division of Geological and Planetary Sciences California Institute of Technology Pasadena CA USA; ^16^ SETI Institute Mountain View CA USA

**Keywords:** mineralogy, diagenesis, Mars, XRD

## Abstract

The *Curiosity* rover's exploration of rocks and soils in Gale crater has provided diverse geochemical and mineralogical data sets, underscoring the complex geological history of the region. We report the crystalline, clay mineral, and amorphous phase distributions of four Gale crater rocks from an 80‐m stratigraphic interval. The mineralogy of the four samples is strongly influenced by aqueous alteration processes, including variations in water chemistries, redox, pH, and temperature. Localized hydrothermal events are evidenced by gray hematite and maturation of amorphous SiO_2_ to opal‐CT. Low‐temperature diagenetic events are associated with fluctuating lake levels, evaporative events, and groundwater infiltration. Among all mudstones analyzed in Gale crater, the diversity in diagenetic processes is primarily captured by the mineralogy and X‐ray amorphous chemistry of the drilled rocks. Variations indicate a transition from magnetite to hematite and an increase in matrix‐associated sulfates suggesting intensifying influence from oxic, diagenetic fluids upsection. Furthermore, diagenetic fluid pathways are shown to be strongly affected by unconformities and sedimentary transitions, as evidenced by the intensity of alteration inferred from the mineralogy of sediments sampled adjacent to stratigraphic contacts.

## Key Points

- CheMin‐determined mineralogy indicates pervasive low‐temperature diagenesis and localized hydrothermal alteration events at Gale crater
- The diagenetic history of Gale crater is characterized by a diverse range in fluid chemistry, Eh, pH, and temperature
- The distribution of SiO_2_ and FeO_T_ among crystalline and amorphous materials constrains the temperature and duration of diagenesis

## 1 Introduction

The Mars Science Laboratory (MSL) rover, *Curiosity*, has been exploring Gale crater since August 2012 with the primary goal of assessing environments that are, or once were, favorable habitats for life (Grotzinger et al., 2012). The diverse mineralogy, stratigraphy, and geomorphic features of Aeolis Palus (northern crater plains) and Aeolis Mons (informally, Mount Sharp) show evidence of environmental and climatic changes in the early Hesperian (Fraeman et al., 2016; Milliken et al., 2010). In particular, the change from phyllosilicate‐bearing sedimentary units at the base of Mount Sharp to sulfate‐bearing sedimentary units higher in the stratigraphy may signify an increase in aridity at ~3.5 Ga (Milliken et al., 2010). This phyllosilicate to sulfate stratigraphy has been recognized from orbital visible/short‐wave reflectance measurements across the planet (e.g., Bibring et al., 2006; Bishop & Rampe, 2016; Bishop et al., 2008; Carter et al., 2015; Deit et al., 2012; Ehlmann et al., 2011; Wiseman et al., 2010), suggesting a global climate change early in Mars' history. *Curiosity* has climbed the lower layers of Mount Sharp, investigating sedimentology, mineralogy, and geochemistry to characterize the environments preserved by the rocks and to evaluate evidence of past habitable conditions and local, regional, or global indicators of climate change.

*Curiosity*'s scientific payload was designed to assess the textural, geochemical, and mineralogical properties of rocks and soils encountered by the rover. Mastcam provides outcrop‐scale images, the Mars Hand Lens Imager (MAHLI), located on the arm of the rover, provides close‐up images with resolutions a high as ~15 μm/px, and the Mars Descent Imager (MARDI), designed to acquire images of the landing site during descent, provides ground imaging at a 1.5 mm/px maximum resolution (Edgett et al., 2012; Malin et al., 2017). This imaging suite allows for the characterization of millimeter‐to‐meter scale sedimentary features identified along the rover's traverse (e.g., Minitti et al., 2019; Sun et al., 2019; Yingst et al., 2013). Complementing the imaging data sets are mineralogical and geochemical analyses by the CheMin (Chemistry and Mineralogy), APXS (Alpha Particle X‐ray Spectrometer), ChemCam (Chemistry and Camera), SAM (Sample Analysis at Mars), and DAN (Dynamic Albedo of Neutrons) instruments. The CheMin X‐ray diffraction (XRD) instrument uses X‐ray crystallography to identify the mineralogy of drilled rocks and scooped loose sediment (Blake et al., 2012). APXS determines the bulk chemistry of Gale crater samples by X‐ray fluorescence (XRF) and particle‐induced X‐ray emission (PIXE) spectroscopy with a spot size of ~1.5 cm in diameter when deployed to contact (Campbell et al., 2012; Gellert et al., 2015; Thompson et al., 2016; VanBommel et al., 2017). The Laser Induced Breakdown Spectroscopy (LIBS) instrument on ChemCam determines the compositions of surface materials with a spot size of a few 100 microns, providing remotely sensed geochemical data from a distance of ~1–7 m (Maurice et al., 2012; Wiens et al., 2012). SAM evolved gas analyses (EGAs) provide information regarding volatile species released upon the thermal decomposition of phases present in analyzed samples (Mahaffy et al., 2012). Lastly, the DAN instrument provides insight into the subsurface composition (e.g., Mitrofanov et al., 2012) and structure (e.g., Gabriel et al., 2018; Mitrofanov et al., 2016). With a ~0.8‐m scale spot size (FWHM) and maximum measurement depths between 45 and 70 cm, DAN detects low‐energy neutrons allowing for changes in geochemistry, particularly changes in H, Cl, and Fe content, to be monitored along *Curiosity*'s traverse (e.g., Gabriel et al., 2018; Litvak et al., 2016; Tate et al., 2015, 2018). APXS, ChemCam, SAM, and DAN data complement mineralogical data and allow the science team to monitor geochemical trends with stratigraphy (e.g., Gellert et al., 2015; L'Haridon et al., 2018; Mangold et al., 2018; Nachon et al., 2017; Tate et al., 2015, 2018).

**Figure 1:**
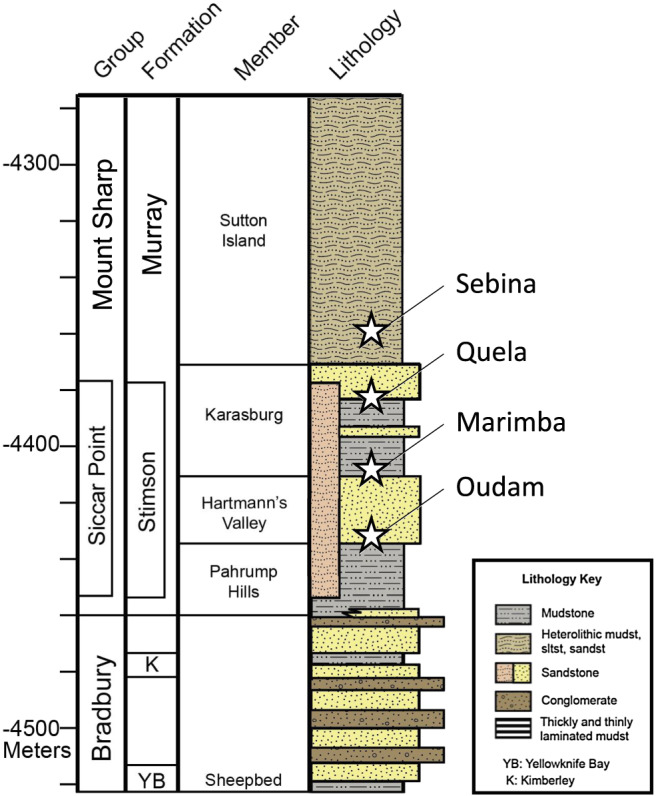
Stratigraphic column of rock units studied by *Curiosity* from landing through Sol 1577. The strata are generally flat‐lying, allowing us to correlate elevation with stratigraphy. Drill hole locations discussed in this manuscript are shown in white stars.

The sedimentology of Gale crater, as determined by *Curiosity*, suggests that Bradbury group rocks and the first ~200 m of the Mount Sharp group were deposited primarily in fluvial‐lacustrine environments (Figure 1). Rivers emanating from the crater rim fed lakes on the crater floor. Conglomerate outcrops near the landing site and sandstone deposits on the plains likely represent fluvial and deltaic deposits, respectively (Grotzinger et al., 2012). The Yellowknife Bay formation within the Bradbury group, studied early in the mission, is dominated by lacustrine mudstone (Grotzinger et al., 2014). CheMin data from two drill samples, John Klein and Cumberland, demonstrate that this mudstone contains silicate minerals characteristic of basalt (i.e., plagioclase, olivine, and pyroxene), minor amounts of magnetite and Ca‐sulfate, and significant proportions of saponite and X‐ray amorphous materials (Vaniman et al., 2014). Upsection from Yellowknife Bay is the Kimberley formation, a unit consisting of fluvial conglomerates, deltaic sandstones, and eolian sandstones. Windjana, the sole drill sample acquired in the Kimberley, is rich in alkali feldspar (interpreted as volcanic; Treiman et al., 2016), unique among all other samples analyzed by CheMin to date, and is one example of the diverse igneous units contributing to Gale crater sediments (Sautter et al., 2015; Treiman et al., 2016). Stratigraphically above the Bradbury group, rocks studied in the Pahrump Hills area represent the lowermost units of the Mount Sharp group and the start of *Curiosity*'s investigation of the Murray formation. The Pahrump Hills member of the Murray formation is composed principally of laminated mudstones, interpreted to represent lacustrine deposits (e.g., Stack et al., 2019). The mineralogy of the Pahrump Hills member is heterogeneous, with primary basalt minerals, smectite, hematite, and jarosite at the base of the unit and greater abundances of plagioclase, crystalline and amorphous silica, and magnetite at the top of the unit (Morris et al., 2016; Rampe et al., 2017). Gradual changes in elemental chemistry are also observed in the Pahrump Hill's section (going upsection, Zn, Ni, and Mn decrease and Ti increases; Rampe et al., 2017). These changes in mineralogy and chemistry have been interpreted as signatures of a stratified lake deposit, a change in sediment source, and/or a diagenetic front (Hurowitz et al., 2017; Rampe et al., 2017).

Here, we focus on the mineralogy and geochemistry of four samples drilled from an 80‐m stratigraphic interval within the Murray formation upsection from the Pahrump Hills. We discuss depositional and diagenetic environments preserved by the members of this interval and compare these members to the mineralogy and geochemistry of previously sampled lacustrine deposits in Yellowknife Bay and Pahrump Hills to describe the depositional and diagenetic history of this region of Gale crater.

### 1.1 Stratigraphy of the Murray Formation

To contextualize the ancient depositional environments of Gale crater, a stratigraphic column was constructed for the rocks along *Curiosity*'s traverse (Figure 1; Fedo et al., 2017; Fedo et al., 2018; Grotzinger et al., 2014, 2015). Elevation estimates unit thickness because of the generally flat‐lying nature of the rocks. The Murray formation, part of the Mount Sharp group, consists primarily of lacustrine mudstones and has, thus far, been divided into seven members (Edgar et al., 2020; Fedo et al., 2018). The lowermost member, the Pahrump Hills, is ~25 m thick and is dominated by finely laminated mudstone. Minor cross‐bedded sandstones, intercalated with the mudstone, suggest a lacustrine to fluvial‐deltaic depositional environment. Overlying the Pahrump Hills member is the Hartmann's Valley member, a ~25‐m‐thick section of cross‐bedded siltstone to fine‐grained sandstone. The scale of observed cross‐stratifications is consistent with sediment transport in eolian or fluvial settings (Fedo et al., 2017; Fedo et al., 2018; Gwizd et al., 2018). Finely laminated mudstone and fine‐grained sandstone form the major lithologies in the ~37‐m‐thick Karasburg member (Fedo et al., 2017). Its sedimentary structures indicate a primarily lacustrine depositional environment. The Sutton Island member is a ~98‐m‐thick interval of heterolithic mudstone‐sandstone strata. Finely laminated mudstone, ripple cross‐laminated siltstone to sandstone, and cross‐stratified siltstone were documented along the rover traverse (Fedo et al., 2017). These features and the rare presence of desiccation cracks suggest a near‐shore lacustrine depositional environment with intermittent exposure events (Stein et al., 2018). The Hartmann's Valley, Karasburg, and Sutton Island members contain centimeter‐scale concretions and veins, an indication of diagenesis (Sun et al., 2019). Furthermore, sulfate enrichments in the Sutton Island member suggest evaporative episodes resulting in high concentrations of brines and potentially marking a transition to arid, sulfate‐dominated conditions (Rapin et al., 2019). The Blunts Point, Pettegrove Point, and Jura members overly the Sutton Island member and comprise a topographically elevated ridge (Vera Rubin ridge [VRR]) that, based on orbital measurements, was identified as enriched in hematite (Fraeman et al., 2013; Morris et al., 2019). In situ observations show that VRR is composed of fine‐grained, planar laminated mudstones with varied geochemistry and diverse mineral assemblages (Fraeman et al., 2020; Morris et al., 2019; Thompson et al., 2019). At the time of writing, the Glen Torridon area, which is clay‐bearing from orbit, is currently being investigated by *Curiosity* (Fox et al., 2020).

## 2 Methods

### 2.1 Sample Selection and Acquisition

**Figure 2:**
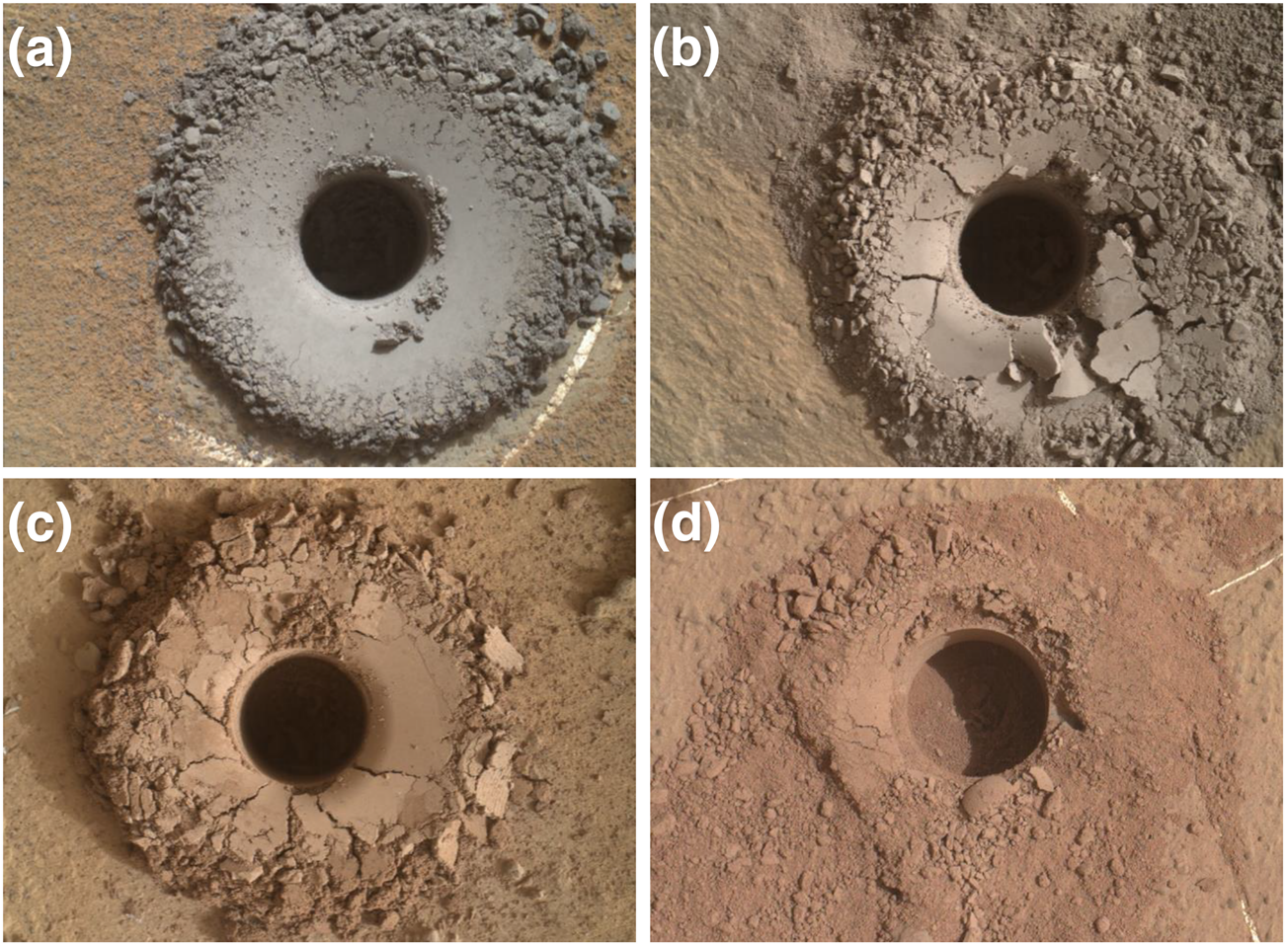
MAHLI images of the drill holes of (a) Oudam; (b) Marimba; (c) Quela; and (d) Sebina.

As part of a systematic drilling campaign to sample the Murray formation every ~25 m of elevation, four drill samples were acquired from strata overlying the Pahrump Hills member: Oudam from the base of the Hartmann's Valley member, Marimba and Quela from the Karasburg member, and Sebina from the Sutton Island member (Figures 1 and 2). Oudam, the first sample of the systematic drilling campaign, was targeted to explore how sedimentary structures, diagenetic textures, geochemistry, and/or mineralogy differ in this orbitally “bright” portion of the Murray formation. The Marimba and Quela targets, 25 m and 56 m stratigraphically above Oudam, respectively, are mudstone samples drilled in the lower and upper regions of the Karasburg member. The Marimba and Quela drill sites represent average Karasburg bedrock at the selected elevations and were analyzed to monitor potential compositional changes arising from diagenetic processes through time. Sebina, located in the Sutton Island member, was acquired 19 m above Quela and was sampled based on observations of bright‐toned terrain from orbit and the presence of nodular features observed in rocks throughout the area.

The Oudam, Marimba, Quela, and Sebina drill samples were acquired on Sols 1361, 1422, 1464, and 1495, respectively. The MSL drill penetrated ~5–6 cm in target rocks and collected the powder for processing, delivery, and analysis by the CheMin and SAM instruments. The upper ~1.5 cm of drilled rock did not enter the auger; the lower ~3–4 cm was transferred up the drill stem to the Collection and Handling for in situ Martian Rock Analysis (CHIMRA) processing system. The drill powder was sieved to <150 μm and portioned for delivery to the CheMin and SAM instruments (Anderson et al., 2012).

### 2.2 Analysis Methods

APXS determines the chemical compositions of rocks and soils by PIXE and XRF spectroscopy and can quantify the abundance of elements with Z > 10 (Gellert et al., 2015). APXS analyses of the Oudam, Marimba, Quela, and Sebina materials most representative of the powders analyzed by CheMin (i.e., drill tailings or piles of sieved sample dumped from CHIMRA) were acquired on Sols 1368, 1426, 1466, and 1496, respectively, and resulting data are listed in supporting information Table S1.

The CheMin X‐ray diffractometer produces diffraction patterns of scooped soils and drilled rock samples (Blake et al., 2012). Drill samples are delivered to sample cells with Mylar or Kapton windows, and a piezoelectric actuator randomizes grain orientations. Diffraction patterns are collected as two‐dimensional (2D) images on a CCD. The 2D images are converted to 1D patterns using a modified version of the GSE_ADA software (Dera et al., 2013), and the data are analyzed with whole pattern fitting FULLPAT (Chipera & Bish, 2002, 2013) and Rietveld refinement methods. XRD analyses provide the proportions of crystalline, clay mineral, and amorphous components, the identity of crystalline phases present at >1 wt%, and unit‐cell parameters for major crystalline phases.

In phyllosilicate‐rich samples, the dioctahedral and/or trioctahedral nature of identified phyllosilicates is estimated from H_2_O thermal decomposition temperature detected by SAM EGA and properties of 02*l* diffraction. If both dioctahedral and trioctahedral forms exist in a single sample, relative ratios are determined by BGMN Rietveld refinement models of the CheMin patterns (see supporting information; Bergmann & Kleeberg, 1998; Ufer et al., 2004). The chemical composition of the amorphous component is estimated from mass‐balance calculations using (1) APXS bulk sample compositions of the drill tailings, (2) the CheMin measured mineral abundances and FULLPAT estimated clay mineral abundances, (3) the crystal chemistries of major phases calculated from unit‐cell parameters (Morrison et al., 2018), (4) the compositions of minor mineral phases from literature values, and (5) clay mineral compositions based on the dioctahedral and/or trioctahedral nature of the observed phyllosilicate and the relative octahedral Fe abundance constrained by SAM EGA traces (see supporting information for details; Morrison, Downs, Blake, Vaniman, et al., 2018). It is important to note that the calculated chemical composition of amorphous components will also include elements associated with crystalline phases below the detection limit of CheMin (approximately <1 wt%) and trace and minor elements associated with substitutional impurities in the CheMin‐detected crystalline phases. The initial CheMin analyses of Oudam, Marimba, Quela, and Sebina were acquired on Sols 1363, 1426, 1472, and 1499, respectively. Four XRD analyses over multiple sols were acquired for each sample totaling 30 analysis hours for Oudam, Marimba, and Quela and 25.5 hr for Sebina.

Over the duration of data collection, variations in the clay mineral abundance relative to crystalline phases were observed in the Marimba XRD patterns; therefore, the reported distribution of crystalline, clay mineral, and X‐ray amorphous phases are derived from the XRD pattern acquired from the first night of analysis. This initial data set is considered most representative of the Marimba drill powder. The dehydration of gypsum to bassanite was observed in all four samples (Vaniman et al., 2018); therefore, the relative abundances of Ca‐sulfate minerals are derived from the first night's XRD pattern and then applied to the distribution derived from the summed pattern (patterns from all four analysis nights). For all samples, plagioclase and hematite unit‐cell parameters were refined, and plagioclase crystal chemistries were calculated from analyses of the summed pattern (Table S2).

Evolved gas analysis as a function of mass/charge ratio (m/z) and temperature from the SAM instrument provides information about thermal decomposition temperatures and thus constrains the identity of volatile‐containing crystalline and amorphous materials (e.g., McAdam et al., 2014; Sutter et al., 2017). A complicating factor is that in complex samples, individual phases can exhibit broad and/or overlapping gas evolutions (e.g., for H_2_O, dehydration of opal at ~400–550°C and nontronite dehydroxylation at ~400–500°C). Total abundances of gases evolved from samples can be quantified using the assumption and methods described by Archer et al. (2013) and Sutter et al. (2017). SAM EGA analyses were measured on Sols 1382, 1443, and 1722 for Oudam, Marimba, and Quela, respectively. EGA data were not acquired for Sebina.

## 3 Results

**Figure 3:**
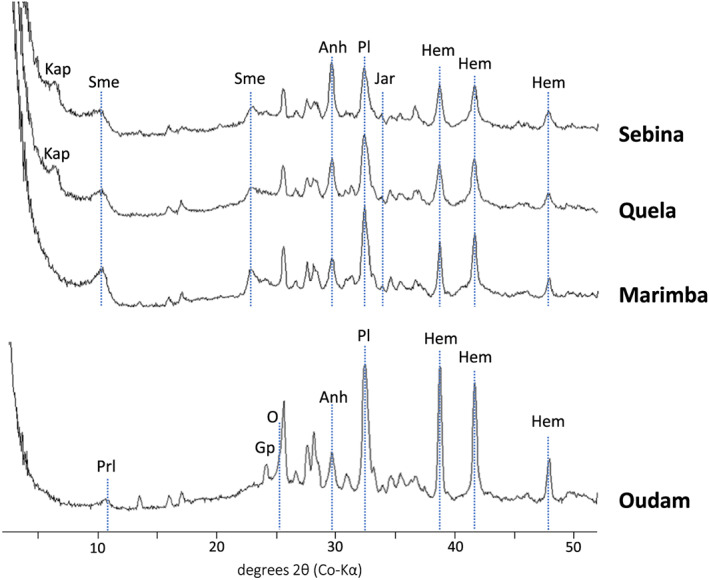
CheMin diffraction patterns of the Oudam sandstone and Marimba, Quela, and Sebina mudstones. Pattern intensities have been normalized to reflect equal analysis durations. Major phases identified are plagioclase (Pl), hematite (Hem), anhydrite (Anh), gypsum (Gp), jarosite (Jar), opal‐CT (O), smectite (Sme), pyrophyllite (Prl), and Kapton (Kap).

The crystalline, clay mineral, and amorphous phase distributions estimated from the diffraction patterns of Oudam, Marimba, Quela, and Sebina drill samples are listed in Table 1, and the patterns are shown in Figure 3. Each sample has between 40 and 60 wt% X‐ray amorphous materials and varying abundances of crystalline and clay minerals (Figure 3 and Table 1). As determined by FULLPAT analyses, Oudam has the highest crystalline fraction (~54 wt%), followed by Marimba, Quela, and Sebina (~32, 32, and 30 wt%, respectively). Clay minerals were identified based on the presence of a broad peak centered near 10 Å and an 02*l* contribution to the pattern at ~22.5 to 23.5 °2θ Co‐Kα. FULLPAT analyses estimate clay mineral abundances between 16 and 28 wt%, for Marimba, Quela, and Sebina, and ~3 wt% is estimated in Oudam.

**Table 1:** Bulk Phase Distributions and Crystalline Phase Abundances for the Oudam Sandstone and Marimba, Quela, and Sebina Mudstones Determined by FULLPAT and Rietveld Analyses, Respectively

	Bulk phase distributions			
	Oudam	Marimba	Quela	Sebina
Crystalline	54	32	32	30
Clay minerals	3(1)	28(3)	16(2)	19(2)
Opal‐CT	7(1)	0	0	0
Amorphous	36(9)	40(10)	52(13)	51(13)
Total	100	100	100	100
	Crystalline mineralogy			
	Oudam	Marimba	Quela	Sebina
Plagioclase	52.3(11)	44.3(30)	43.6(33)	37.9(22)
Hematite	25.6(7)	20.4(14)	22.7(17)	23.7(23)
Pyroxene	8.1(17)	2.2(19)	7.3(19)	6.9(38)
Anhydrite	6.4(5)	11.7(14)	9.4(5)	16.9(10)
Gypsum	5.8(4)	6.6(9)	1.3(1)	3.5(2)
Quartz	1.8(4)	1.7(6)	1.1(7)	0.9(2)
Sanidine	—	7.5(18)	6.5(13)	4.2(13)
Bassanite	—	3.9(10)	5.9(3)	3.5(2)
Jarosite	—	1.7(8)	1.4(5)	2.5(5)
Halite	—	—	0.8(2)	—
Total	100	100	100	100

*Note*. Abundances in wt%; uncertainties reported as 1σ.

### 3.1 Crystalline Component

The crystalline phases identified in the Oudam, Marimba, Quela, and Sebina samples are listed in Table 1. Plagioclase is the dominant mineral in each sample followed by hematite and gypsum. The calculated composition of plagioclase, based on refined unit‐cell parameters (Morrison, Downs, Blake, Vaniman, et al., 2018), is similar in all samples: An40(7) for Oudam, An39(5) for Marimba, An39(6) for Quela, and An42(6) for Sebina (Table S2). The plagioclase compositions are within uncertainty of the average composition, An38(2), calculated for the four Pahrump Hills member drill samples (Morrison et al., 2018; Rampe et al., 2017). Hematite is the second most abundant mineral in each drill sample, followed by crystalline Ca‐sulfates. Oudam has the highest abundance of hematite among the four samples. Gypsum, bassanite, and anhydrite were detected in Marimba, Quela, and Sebina, whereas only gypsum and anhydrite were detected in Oudam. Minor phases present in all four drill samples are pyroxene and quartz. Minor amounts of jarosite and sanidine were identified in Marimba, Quela, and Sebina, and trace amounts of halite were identified in Quela.

### 3.2 Clay Minerals

**Figure 4:**
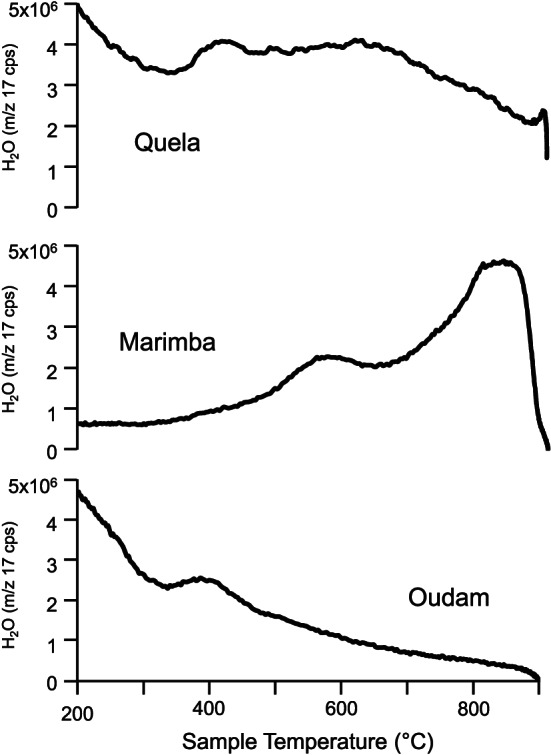
SAM EGA H_2_O profiles for Oudam, Marimba, and Quela.

Clay minerals are present in all four drill samples. The Oudam diffraction pattern shows a broad, low intensity diffraction peak at ~9.6 Å, assigned to the (001) basal diffraction of a phyllosilicate mineral. This (001) peak position is smaller than any previously observed in CheMin (~10 Å; Bristow et al., 2018 ; Rampe et al., 2017 ; Vaniman et al., 2014). The position and breadth of the Oudam basal peak are consistent with zero‐layer charge 2:1 dioctahedral phyllosilicates (e.g., pyrophyllite; Bristow et al., 2018), acid‐altered smectites (e.g., Craig et al., 2014), and low‐charge, zero‐hydrate smectites (e.g., Moore & Hower, 1986). The SAM EGA profile shows a single H_2_O release at ~410°C, attributed to the dehydroxylation of a dioctahedral phyllosilicate (Figure 4).

**Figure 5:**
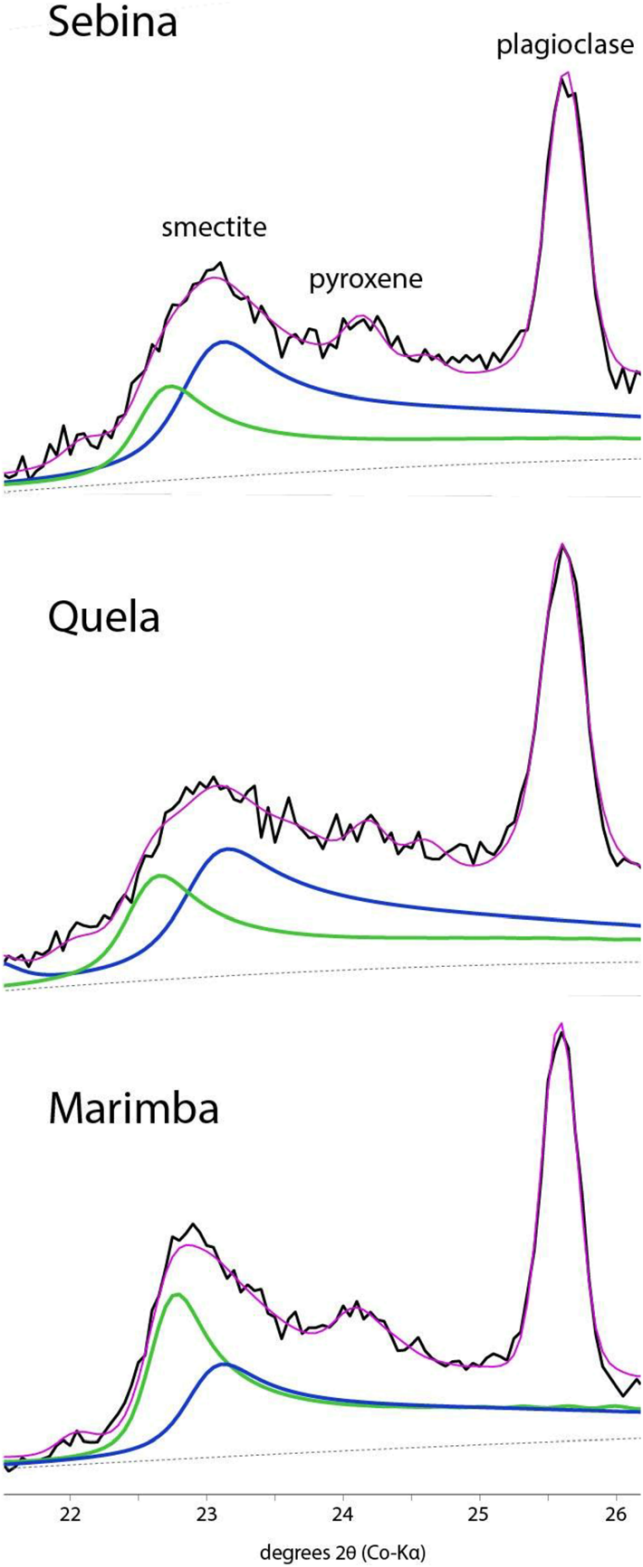
BGMN models of dioctahedral (blue) and trioctahedral (green) smectite profiles for Marimba, Quela, and Sebina 02*l* smectite bands. Data are available through Bristow et al. (2018).

Basal clay mineral peaks observed in Marimba, Quela, and Sebina diffraction data are similar to those in the Yellowknife Bay and Pahrump Hills mudstones (Bristow et al., 2018; Rampe et al., 2017; Vaniman et al., 2014), namely, a broad diffraction peak at ~10 Å and a 02*l* band near ~4.50 Å consistent with collapsed smectites. The highest smectite abundance (~28 wt%) is in the Marimba drill sample (Table 1). BGMN models of the Marimba 02*l* band are consistent with a 1:2 dioctahedral:trioctahedral (di:tri) smectite weight abundance ratio (Figure 5; Bristow et al., 2018). Two H_2_O releases at 610°C and 825°C are present in the Marimba EGA trace reflecting dehydroxylation temperatures of both dioctahedral and trioctahedral phyllosilicates (Figure 4). The 610°C and 825°C dehydroxylations are most consistent with an Fe‐montmorillonite and an Mg‐saponite, respectively (McAdam et al., 2017).

The Quela and Sebina XRD patterns have clay mineral scattering profiles similar to Marimba (i.e., broad basal peaks at ~10 Å and 02*l* bands at ~4.50 Å). A BGMN model of the Quela diffraction data results in a 1:1 di:tri smectite estimate, and modeling of the Sebina clay profile yields a di:tri smectite ratio of 5:3 (Figure 5). The SAM EGA profile of Quela shows an H_2_O release ~470°C, a broad H_2_O release between 550°C and 740°C, and a sharp H_2_O release at ~835°C (Figure 4). The high temperature release is attributed to a trioctahedral smectite, and the wide, mid‐temperature evolution can be attributed to a dioctahedral smectite and/or an opaline‐SiO_2_ phase. The ~470°C evolution is consistent with jarosite and Fe‐rich phyllosilicates (McAdam et al., 2017; Sutter et al., 2017).

### 3.3 Amorphous Materials

The diffraction patterns for Oudam, Marimba, Quela, and Sebina show low‐angle scattering (elevated intensity with decreasing 2θ) and an elevated background, indicative of the samples containing X‐ray amorphous materials. The prominent amorphous hump in Oudam is centered at a lower 2‐theta angle (~25 °2θ, Co‐Kα) versus Marimba, Quela, and Sebina (~30, ~29, ~29 °2θ, Co‐Kα, respectively). Shifts toward lower 2‐theta angles are consistent with more Si‐rich amorphous materials and are supported by the estimated composition of the Oudam amorphous component (see discussion below). The calculated compositions of amorphous materials in each drill sample are listed in Table 2.

**Table 2:** Calculated Composition of the Amorphous Component Presented as the Abundance Estimated in the Bulk Sample and as the Percentage of the Amorphous Fraction

	Bulk sample				Amorphous fraction			
	Oudam^a^	Marimba	Quela	Sebina	Oudam	Marimba	Quela	Sebina
SiO_2_	31.7	23.5	23.2	25.3	64.3	44.5	44.9	49.5
TiO_2_	1.0	1.0	1.0	1.1	2.1	2.0	2.0	2.1
Al_2_O_3_	2.7	2.6	2.0	2.1	5.4	4.9	3.8	4.2
Cr_2_O_3_	0.3	0.3	0.3	0.3	0.6	0.6	0.6	0.6
FeO_T_	5.5	16.8	10.5	9.7	11.2	31.7	20.4	19.0
MnO	0.2	0.1	0.2	0.1	0.5	0.1	0.4	0.3
MgO	4.1	0.0	1.2	1.6	8.3	0.0	2.2	3.1
CaO	0.0	1.9	3.9	2.9	0.0	3.6	7.6	5.7
Na_2_O	0.8	1.0	0.9	1.0	1.7	2.0	1.7	2.1
K_2_O	0.8	0.5	0.4	0.6	1.7	1.0	0.8	1.1
P_2_O_5_	0.5	1.0	1.1	0.6	1.0	2.0	2.1	1.2
SO_3_	1.2	3.6	6.1	4.7	2.5	6.8	11.7	9.3
Cl	0.3	0.5	0.9	1.0	0.7	0.9	1.7	2.1
Total	49.1^b^	52.9^b^	51.5^c^	51.1^c^	100	100	100	100

*Note*. Oxides and Cl are reported in wt%.

^a^ Opal‐CT considered an amorphous phase.

^b^ APXS‐constrained amorphous abundance.

^c^ FULLPAT‐derived amorphous abundance.

SiO_2_ is the dominant oxide in each sample's amorphous component. Oudam has the largest abundance of amorphous SiO_2_, a portion of which is attributed to opal‐CT. High SiO_2_ abundances interpreted as opaline silica were also observed in alteration halos in the Stimson sandstone unit (~64 and ~68 wt% for Greenhorn and Lubango samples, respectively; Yen et al., 2017), a unit that uncomformably overlies the Murray, as well as the Buckskin mudstone (~77 wt%; Morris et al., 2016; Rampe et al., 2017) approximately 11 m below the Oudam drill sample (Figure 1). The inference of opaline silica and stratigraphic relationship among the Greenhorn, Lubango, Buckskin, and Oudam drill samples may be linked to diagenetic processes (see below).

**Figure 6:**
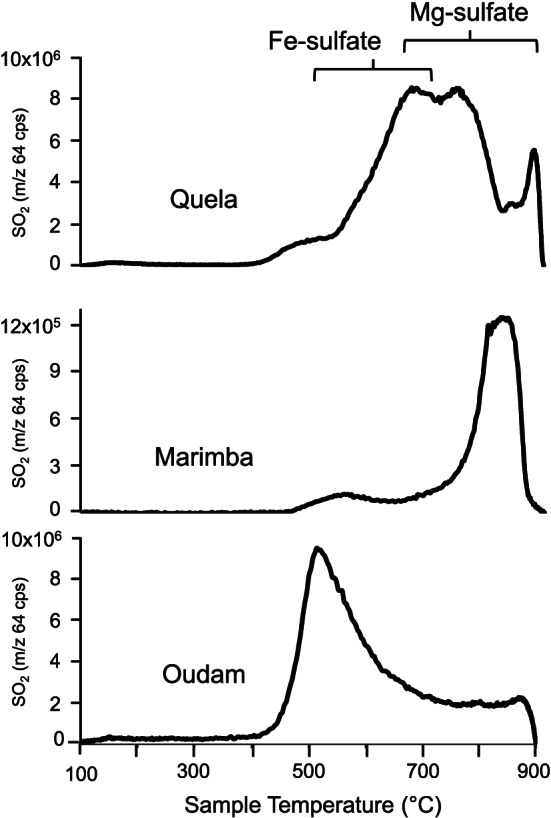
SAM EGA profiles for evolution of SO_2_ with temperature.

FeO_T_ is the second‐most abundant oxide in the amorphous component (Table 2). Marimba has the highest abundance of FeO_T_ in the amorphous fraction (~17 wt% among all samples analyzed to date), and SO_3_ is significant in the amorphous fraction of Quela and Sebina. Even though the bulk SO_3_ is higher for Quela and Sebina, the fraction attributed to the crystalline phases (Ca‐sulfates) is similar among all four samples. The additional SO_3_ detected in Quela and Sebina is attributed to amorphous S‐bearing phases. SAM EGA profiles for Oudam, Marimba, and Quela show SO_2_ evolutions at temperatures >500°C, consistent with Fe‐sulfates (~500–700°C) and Mg‐sulfates (>700°C) (Figure 6; Mcadam et al., 2014; Sutter et al., 2017). SO_3_ abundances estimated from SO_2_ EGA profiles are 0.3, 0.2, and 3.4 wt% SO_3_ for Oudam, Marimba, and Quela, respectively (Sutter et al., 2019). Sulfides are unlikely components of these samples as SO_2_ evolutions below 500°C, consistent with sulfide oxidative decomposition, were not observed (Sutter et al., 2017). Sulfur and other S‐bearing species could also contribute to the SO_2_ evolutions observed; however, because they were not detected by CheMin, such phases would be present below the instrument detection limits or X‐ray amorphous (McAdam et al., 2014; Rampe et al., 2016).

## 4 Discussion

The crystalline and clay minerals in Oudam, Marimba, Quela, and Sebina are consistent with exposure to multiple episodes of aqueous alteration. In conjunction with sedimentological observations, mineralogical and geochemical data from the Hartmann's Valley, Karasburg, and Sutton Island members allow us to assess the depositional history of these sedimentary rocks and speculate how changes in sediment source, lake water conditions, and groundwater influx produced the mineralogy and geochemistry of the rock samples analyzed by *Curiosity*.

### 4.1 Oudam—Hartmann's Valley Member

The mineralogy and sedimentology of the Hartmann's Valley member are unique within the Murray formation and make a definitive assessment of this unit's depositional history challenging. Meter‐scale trough cross‐bedding suggests sand dunes, in either eolian or fluvial environments (Gwizd et al., 2018). Sands from these two depositional settings typically have distinct grain size distributions. Close inspection of Hartmann's Valley MAHLI images show that individual sand grains cannot be resolved in ~75% of the total imaging area (Gwizd et al., 2018). These ambiguous regions possess either grains smaller than the image resolution (~17 μm/px) or larger grains whose boundaries are masked by secondary effects like abrasion, compaction, or diagenesis (Gwizd et al., 2018). Resolvable grains in the MAHLI images appear to be primarily coarse silt to fine sand (~30–125 μm), a size distribution smaller than that of saltating grains in martian eolian dunes (~100–250 μm) and smaller than grains predicted for a martian fluvial dune setting (>75 μm) (Cousin et al., 2015; Grotzinger et al., 2013; Gwizd et al., 2018). In the absence of a definitive paleoenvironment determination, we discuss the mineralogy of Oudam in the contexts of both eolian and fluvial environments to infer the detrital source of the sediments and constrain the diagenetic conditions that resulted in the observed mineral assemblage.

The detrital minerals recognized in Oudam are plagioclase and pyroxene, which suggest a basaltic parent material. The absence of olivine, coupled with the high plagioclase to pyroxene ratio (~5.2 compared to ~1.4 for Gale soils and Stimson unaltered sandstones), indicates that the parent material was depleted in olivine and pyroxene relative to other Gale eolian samples or that Oudam experienced a higher degree of aqueous alteration. Based on the assemblage of secondary minerals in Oudam, we prefer the latter scenario and argue this alteration occurred diagenetically (see below).

Secondary minerals observed by CheMin and SAM in Oudam include Ca‐, Mg‐ and Fe‐sulfates, hematite, opal‐CT, and a 9.6 Å phyllosilicate. Oudam is enriched in Ca‐sulfate compared to mudstones sampled in the Pahrump Hills and Yellowknife Bay formations and the unaltered sandstones analyzed in the Stimson formation. Veinlets were observed adjacent to the Oudam drill site, a feature attributed to a late‐stage diagenetic event; however, the lack of veinlets observed in the Oudam borehole suggests that the Ca‐sulfates detected in CheMin diffraction data could be present in the sedimentary matrix as cement. In addition to Ca‐sulfates, SAM EGA profiles indicate the presence of Fe‐ and Mg‐rich sulfates. The Fe‐ and Mg‐sulfate phases are interpreted as X‐ray amorphous or, if crystalline, present below the CheMin detection limit (<1 wt% crystalline).

Hematite is abundant in the Oudam drill sample. Images of the Oudam borehole and tailings are gray in color (Figure 2a), suggesting that hematite crystallites in Oudam are >5 μm (Lane & Christensen, 1999), thus restricting the conditions under which the hematite precipitated. Two pathways may be responsible for the formation of sedimentary, gray hematite, (1) oxidation and precipitation of iron oxides in a low‐temperature standing body of water followed by thermal recrystallization upon burial, or (2) formation under hydrothermal conditions (e.g., Catling & Moore, 2003).

A standing body of water, described in the first scenario, is inconsistent with an eolian or fluvial setting, so the mechanisms associated with burial and thermal recrystallization should be explored. Similar to the oxidative diagenesis described for Stimson sandstones (Yen et al., 2017), dissolution of olivine and pyroxene to produce Fe‐oxides during burial/lithification is a probable scenario for the Oudam sandstones. To achieve complete oxidation and recrystallization to gray hematite, ~3–5 km of burial depths would be required to reach the required formation temperature (>100°C; Catling & Moore, 2003). Maximum burial depths for Gale crater sediments are estimated between 1.6 and 1.8 km assuming poorly lithified, fine‐grained silts (Lewis et al., 2019). Greater burial depths result if the porosity of Oudam sediments was lower than the 40% assumed in the Gale crater models. However, we do not expect depths in the ~3–5 km range as this would also affect the expression of hematite in adjacent sediments like Marimba, where amorphous FeO_T_ is abundant and the red‐colored drill fines suggest hematite crystallites <5 μm. Therefore, while Fe‐oxides were likely produced during burial, an additional source of heat is required for the formation of gray hematite in Oudam.

A second pathway to form gray hematite, hydrothermal alteration of iron‐oxides, is our preferred formation mechanism for the hematite observed at Oudam. At elevated temperatures (>100°C), precursor Fe‐oxides (e.g., magnetite, FeOOH polymorphs, and small hematite crystallites) transform to gray hematite. For example, hematite can result from the thermal oxidation of magnetite (variety martite), dissolution and reprecipitation of goethite, rearrangement of ferrihydrite, and Ostwald ripening of hematite crystallites. If the hematite formed from an Fe^2+^‐bearing precursor, H_2_O and/or sulfate, in sulfate‐rich waters, act as the oxidant, resulting in H_2_ and/or sulfide production. Although sulfides were not identified in diffraction data, a negative δ^34^S isotope composition was measured for Oudam and interpreted to represent sedimentary sulfides (Franz et al., 2017). If sulfides were a product of this hydrothermal redox event, the δ^34^S signature recorded would remain unchanged even upon subsequent oxidation of the sulfides during a later diagenetic event. We cannot exclude the possibility of detrital sulfides; however, all previous drill samples (excluding Cumberland) exhibit δ^34^S values consistent with sulfates/sulfites (Franz et al., 2017).

The presence of a 9.6 Å phyllosilicate and amorphous SiO_2_ provide additional constraints on diagenetic events recorded in the Oudam sample. The phyllosilicate could be detrital or produced in situ through aqueous alteration processes. If detrital, the phyllosilicate was likely a component of the windblown sand, originating from a local or regional source containing low‐charge smectite, hydrothermally generated pyrophyllite, or acidically altered nontronite. If diagenetic, a windblown, detrital smectite later exposed to acidic fluids could result in an altered smectite structure, reducing the basal spacing from ~10 to ~9.6 Å (e.g., Craig et al., 2014). Alternatively, Fe‐pyrophyllite may form in moderately warm fluids (as low as ~55–65°C; Badaut et al., 1992), a scenario that could link the phyllosilicate and gray hematite to the same hydrothermal event.

The Oudam diffraction pattern also exhibits evidence of opal‐CT, and the calculated amorphous composition is enriched in silica compared to other CheMin‐analyzed samples analyzed (excluding Buckskin and Stimson alteration halo samples; Rampe et al., 2017; Yen et al., 2017). Dissolution of Fe‐Mg‐silicates often results in the precipitation of Fe‐oxides and a silica‐rich residue (e.g., McLennan, 2003). Subsequent diagenesis of amorphous silica (i.e., opal‐A) may result in the formation of opal‐CT through dissolution and reprecipitation reactions (e.g., Botz & Bohrmann, 1991; Kastner et al., 1977; Williams & Crerar, 1985). Maturation of opal‐A to opal‐CT has been reported at various temperatures, most commonly between ~20°C and 60°C (~e.g., Hein et al., 1978; Isaacs et al., 1983; Murata et al., 1977; Pisciotto, 1981). If the quartz observed in Oudam were not detrital but resulted from the maturation of opal‐CT, temperatures upward of 110°C would be required to facilitate the transformation (e.g., Pisciotto, 1981).

Evidence for warm fluids at Oudam is supported by observations of gray hematite and opal‐CT, and the possibility of Fe‐pyrophyllite. To bound the hydrothermal temperatures proposed at Oudam, both thermal models and time of alteration must be considered. The most common gray hematite formation mechanisms suggest minimum temperatures >80°C. These temperatures are within the opal‐CT to quartz transformation range and near the upper limit of observed opal‐A to opal‐CT transformation temperatures. In addition to temperature, multiple factors influence opal‐CT formation (e.g., permeability, fluid chemistry, and pH); however, most of these parameters are unknown or poorly constrained at Oudam (Williams & Crerar, 1985). The coexistence of opal‐A, opal‐CT, quartz, and gray hematite suggests multiple fluid events influenced the final Oudam mineral assemblage. Moderate temperatures would facilitate opal‐CT formation with subsequent transformation to quartz and formation of gray hematite during a warmer event (>100°C). The persistence of pyroxene implies that the duration of these aqueous diagenetic events was likely limited and/or water‐to‐rock ratios were low.

Elevated temperatures can be attributed to impact‐derived hydrothermal systems, burial diagenesis, or magmatic activity. Impact studies suggest that hydrothermal systems may remain active for ~380,000 years following the creation of a 180‐km‐diameter crater (Gale crater ~150 km) and relic hydrothermal flow and weakly circulating hydrothermal systems can persist up to 2 Ma after impact (Abramov & Kring, 2005). If the fluids at Oudam were warmed by impact‐related processes, deposition, lithification, and erosion of Murray rocks and the deposition and lithification Stimson formation rocks are required within ~0.38–2 Ma following the Gale impact. Burial diagenesis models predict temperatures upward of 125°C for Gale crater sediments (Borlina et al., 2015). If gray hematite was observed throughout Hartmann's Valley, burial diagenesis is a plausible mechanism for achieving elevated temperatures; Oudam was the only drill sample in this stratigraphic unit, so we cannot test this hypothesis. Furthermore, gray hematite is not pervasive throughout the Murray formation below Hartmann's Valley, which we would expect if warm, burial‐associated diagenetic fluids were responsible for the gray hematite. However, heat generated by burial and compaction of Gale sediments could cause warm fluids from deep regions of the basin to remobilize and migrate upward. Lastly, evidence of volcanism within the crater or nearby has not been observed; however, groundwater heating by shallow magmatic activity is a plausible mechanism to generate and circulate hydrothermal fluids. Lacking both the time required to achieve complete lithification of both Murray and Stimson rocks and evidence for widespread gray hematite in units below Oudam, we prefer near‐surface magmatism or a deep basin reservoir as the source of hydrothermal fluids at Oudam and believe the Murray‐Stimson contact strongly influenced the path of migration.

**Figure 7:**
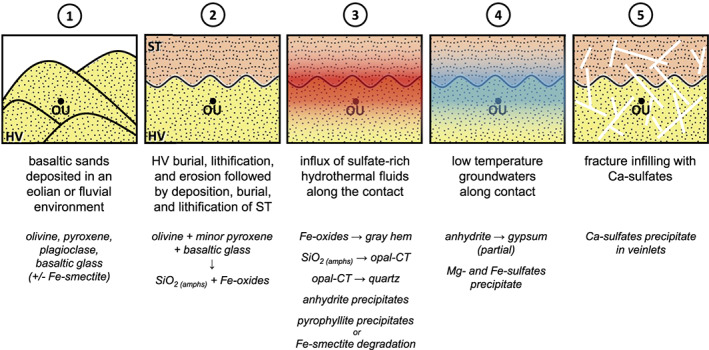
Major events in the depositional and diagenetic history of rocks at the Oudam drill site. (1) Deposition of Hartmann's Valley (HV) basaltic sands; (2) lithification/burial resulting in dissolution of Fe‐Mg‐silicates to form Fe‐oxides and amorphous silica, erosion of HV, and deposition lithification/burial of Stimson (ST) sands; (3) hydrothermal fluids flow along the HV/ST contact causing dissolution of amorphous silica and reprecipitation as opal‐CT, Fe‐oxide/oxyhydroxide precipitation and/or recrystallization to gray hematite, formation of minor Fe‐pyrophyllite (or degradation of nontronite), and precipitation of anhydrite; (4) influx of low‐temperature groundwaters resulting in partial rehydration of anhydrite to gypsum and precipitation of minor Mg‐ and Fe‐sulfates; and (5) veinlet formation due to late‐stage fracturing and subsequent infilling by Ca‐sulfates can be concurrent with Step 4 if fractures were the result of volume expansion induced by the partial rehydration of anhydrite to gypsum. See Figure 1 for lithology legend.

Based on the mineral assemblage observed for Oudam, we propose the following scenario (Figure 7), (1) deposition of basaltic sands in an eolian and/or fluvial environment, (2) lithification/burial resulting in dissolution of Fe‐Mg‐silicates to form Fe‐oxides and amorphous silica, (3) hydrothermal fluids interact with the rock causing dissolution of amorphous silica and reprecipitation as opal‐CT and quartz, Fe‐oxide/oxyhydroxide recrystallization to gray hematite, formation of minor Fe‐pyrophyllite (or degradation of smectite if fluids were acidic), and precipitation of anhydrite, (4) influx of low‐temperature groundwaters inducing partial rehydration of anhydrite to gypsum and precipitation of minor Mg‐ and Fe‐sulfates, and (5) late‐stage fracturing and subsequent fracture infilling by Ca‐sulfates (may occur concurrent with Step 4).

### 4.2 Marimba, Quela, and Sebina—Karasburg and Sutton Island Members

**Figure 8:**
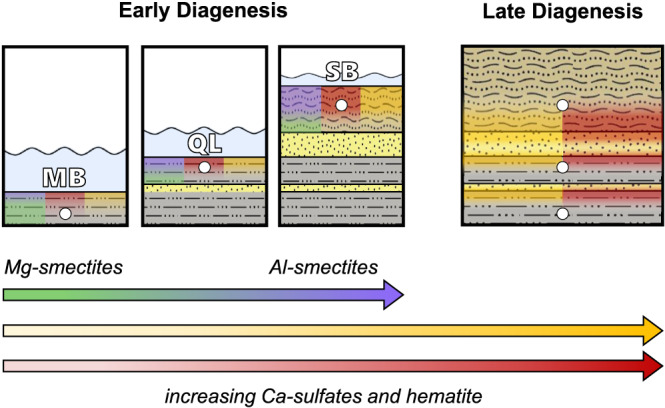
The depositional and diagenetic history of rocks at Marimba, Quela, and Sebina. Basaltic sediments are deposited in a lacustrine environment resulting in the aqueous alteration of Fe‐Mg‐silicates to smectite and Fe‐oxides. Sediments are further altered due to episodic shallowing and evaporative events that increase over time. As a result, early diagenesis is marked by an increase in cation mobility and more oxic waters with higher sulfate concentrations. Late diagenesis is characterized by the influx of oxic and sulfate‐rich groundwaters (possibly associated with lithology transitions), augmenting the Ca‐sulfate and hematite abundances. Combined, early and late diagenesis resulted in an increase in Al‐smectites, Ca‐sulfates, and hematite from Marimba to Sebina. See Figure 1 for lithology legend.

The mineralogy, geochemistry, and sedimentary features observed in the Marimba, Quela, and Sebina mudstone samples and the surrounding strata suggest deposition in lacustrine and marginal‐lacustrine environments, with episodes of desiccation, followed by later diagenetic events (Figure 8). Plagioclase, clay minerals, Ca‐sulfates, and hematite are the most abundant crystalline phases detected by CheMin. Marimba had the largest clay mineral proportion detected in Gale crater prior to analyses obtained from Glen Torridon samples, with a dioctahedral:trioctahedral ratio of 1:2 (Bristow et al., 2018, 2019; Rampe et al., 2017; Vaniman et al., 2014). The magnesian nature of Marimba smectites is consistent with the absence of olivine and low proportions of pyroxenes detected in the sample, likely precursors to the trioctahedral smectite and indicative of a basaltic provenance. The coexisting dioctahedral smectite is believed to have formed under oxidative, open‐system aqueous alteration, depleting Mg and enriching Al (Bristow et al., 2018). Smectites in Quela and Sebina are less abundant than in Marimba, possibly due to less extensive alteration of mafic phases, as shown in the higher abundances of pyroxene compared to Marimba. Alternatively, the Quela and Sebina detrital source may have possessed a lower olivine:pyroxene ratio, thus limiting smectite formation.

The presence of both trioctahedral and dioctahedral smectites in the Marimba, Quela, and Sebina samples indicates depositional conditions unlike those inferred for Yellowknife Bay or the Pahrump Hills. The presence of trioctahedral smectites implies alteration of mafic phases (i.e., olivine and pyroxene) to saponite or indicates more magnesian water chemistries, resulting from evaporative episodes, near the time of deposition (Bristow et al., 2018). Dioctahedral smectites likely formed in an open‐system aqueous environment with episodic evaporation, resulting in the mobilization of elements, oxidation of sediments, and subsequent formation of the Al‐rich smectites (Figure 8; Bristow et al., 2018). Increasing di:tri ratios (1:2, 1:1, and 5:3 for Marimba, Quela, and Sebina, respectively) going upsection indicate decreased Mg^2+^ activities in pore or lake waters, or enhanced rates of element mobility during early diagenesis. Sedimentological observations of increased grain size from Marimba to Sebina could have promoted minor late‐stage diagenetic alteration of trioctahedral to dioctahedral smectite resulting from higher permeabilities in adjacent rocks that could permit greater fluid flow and element mobilization to and from the mudstones. Alternatively, the observed smectites could be detrital and represent chemical weathering products from a source region supplying sediment to the lake. In this scenario, the variable di:tri ratios could reflect changes in weathering signatures from the source or along the fluid pathway, or different mixtures of detrital and authigenic clays (e.g., Chemtob et al., 2015, 2017; Mangold et al., 2018).

Diagenetic processes had a significant impact on the Karasburg and Sutton Island drill samples, with respect to both mineralogy and compositions of their X‐ray amorphous components. Concretion‐rich rocks, indicative of post‐depositional alteration processes, are present throughout the Karasburg and Sutton Island members (Sun et al., 2019). Crystalline Ca‐sulfates, amorphous sulfates, and hematite are considered products of diagenesis. The lack of sedimentary features supporting syndepositional sulfate formation (e.g., Ca‐sulfate crystal molds, disrupted mud laminations, and enterolithic folding) indicates post‐depositional precipitation of Ca‐sulfates in the sediments. The lack of distinct fracture‐filled veins in the sampled mudstones suggests that Ca‐sulfates are primarily intergranular components; the large hematite fraction is also considered a matrix constituent. Oxic environments supporting hematite and Ca‐sulfate precipitation may have occurred during episodic evaporation events (e.g., Bristow et al., 2018), a prolonged shallow lake setting (e.g., Hurowitz et al., 2017), or exposure to oxic groundwaters. The red color of the Marimba, Quela, and Sebina drill tailings suggests that the hematite in these samples is not as coarsely crystalline (i.e., <5 μm) as the hematite in Oudam. This, along with the lack of opal‐CT, suggests that these samples did not experience diagenesis at elevated temperatures. Lastly, jarosite, a mineral formed in moderately acidic fluids, is observed as a minor phase in the Marimba, Quela, and Sebina drill samples and interpreted as a late‐stage diagenetic product of brief or local acid‐sulfate alteration.

### 4.3 X‐Ray Amorphous Component—Oudam, Marimba, Quela, and Sebina

All Murray formation samples contain significant proportions of X‐ray amorphous material. The inferred compositions of these amorphous materials are diverse and reveal subtle aqueous alteration histories not apparent in their bulk compositions and mineral proportions. Although XRD data alone cannot provide definitive identification of amorphous materials, it in combination with calculated chemical compositions, SAM EGA profiles, and elemental trends enables deeper insights into the characteristics of specific amorphous phases. The occurrence of certain amorphous materials may have implications for source sediment variability and the chemistry, salinity, pH, and/or redox conditions of lacustrine and diagenetic fluids.

**Figure 9:**
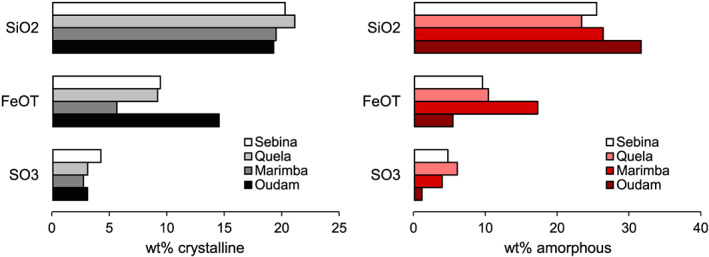
Distribution of major oxides in the crystalline (left) and amorphous (right) components for Oudam, Marimba, Quela, and Sebina.

Variations in the distribution of major elements among crystalline and amorphous materials are depicted in Figure 9. One of the most significant differences among amorphous components in the four drill samples is the abundance of SiO_2_ in the Oudam amorphous fraction. The Buckskin mudstone and Greenhorn and Lubango alteration halos are the only three Gale drill samples with higher abundances of amorphous SiO_2_ than Oudam. The multiple diagenetic episodes proposed for Stimson halos (Yen et al., 2017) and provenance and/or hydrothermal history of Buckskin (Morris et al., 2016; Yen et al., 2018) correlate with the multiple diagenetic episodes proposed for Oudam. Excluding the opal‐CT in Oudam, the amorphous SiO_2_ observed in all four drill samples is consistent with any combination of opal‐A, SiO_2_‐rich glass, amorphous aluminosilicates, and amorphous Fe‐silicates. The presence of abundant amorphous SiO_2_ but absence of maturation to opal‐CT further illustrates low‐temperature diagenesis at Marimba, Quela, and Sebina, compared to Oudam.

The second‐most significant oxide contribution to the amorphous component is FeO_T_. Approximately 75% of the total FeO_T_ observed in Marimba is attributed to the amorphous fraction, greater than any other sample drilled in Gale crater to date. The relatively low abundance of amorphous Fe‐sulfates observed in the Marimba EGA profile (Figure 6; 0.2 wt% SO_3_, Sutter et al., 2019) suggests that nanophase Fe‐oxides and/or amorphous Fe‐silicates are the primary Fe‐bearing amorphous materials. Even though bulk FeO_T_ measured for Marimba is only ~3 wt% greater than that in Oudam, Quela, and Sebina (Table S1), the skewed distribution of FeO_T_ in amorphous phases implies a distinct alteration history among the four drill samples. We attribute this compositional difference to several variables. First, episodic wetting and drying episodes were likely more common in the histories of Quela and Sebina. Any Fe‐oxide produced as a result of saponitization reactions in Quela and Sebina (see Bristow et al., 2015) was (1) not as abundant due to a lower abundance of saponite compared to Marimba and (2) more readily removed due to a higher degree of element mobilization driven by fluctuations in the water table. If the Marimba amorphous Fe‐oxides were subjected to diagenetic events similar to those proposed for Oudam, a more complete maturation to hematite would be expected. The proportion of FeO_T_ attributed to the crystalline fraction in Oudam (~70%) compared to the Marimba, Quela, and Sebina mudstones (26%, 44%, and 47%, respectively) suggests that Marimba, as well as the Quela and Sebina mudstones, experienced fewer and/or lower temperature diagenetic events compared to Oudam.

The last major oxide to consider, SO_3_, increases in bulk abundance upsection from Oudam to Sebina (Table S1). The fraction attributed to crystalline Ca‐sulfates is relatively consistent among the drill samples, whereas the proportion of SO_3_ in the amorphous component increases upsection (Figure 7). SAM SO_2_ EGA profiles show release temperatures consistent with both Fe‐rich and Mg‐rich sulfates (Figure 6). Fe‐rich sulfate phases are dominant in the Oudam SO_2_ profile and are attributed primarily to amorphous Fe‐sulfate material, although minor jarosite may be present below the CheMin detection limit. Deconvolution of the Oudam SO_2_ EGA profile yields ~0.3 wt% SO_3_ (Sutter et al., 2019). Excluding any high temperature (>800°C) amorphous sulfates not detectable by SAM (e.g., Ca‐ and Na‐sulfates), the low abundance of amorphous Fe‐sulfates suggests that these materials are residual precipitates during late‐stage diagenesis. Mg‐rich sulfate is identified at higher abundances in the Marimba and Quela EGA profiles compared to Fe‐rich sulfates. In Marimba, the minor Fe‐sulfate SO_2_ evolution is attributed to jarosite or jarosite plus amorphous Fe‐rich sulfates. A combination of jarosite and amorphous Fe‐sulfates is likely in Quela, evidenced by a prominent SO_2_ evolution at ~700°C. EGA data were not acquired for Sebina, but similarities in mineralogy as well as bulk and amorphous chemistries to Quela imply the presence of both Fe‐ and Mg‐rich sulfates. Mg‐ and Fe‐sulfates may have formed in the lake waters upon dissolution of mafic phases or during a diagenetic event. Approximately 0.2 and 3.4 wt% SO_3_ in Marimba and Quela, respectively, are estimated from SAM SO_2_ EGA data (Sutter et al., 2019). The higher jarosite abundances observed in Quela versus Marimba cannot solely account for the increase. Coupled with the increasing di:tri smectite ratio, Fe and Mg ions were likely elevated in Quela fluids, possibly supporting higher abundances of Fe‐ and Mg‐sulfate precipitation in late‐stage diagenetic events. The presence of jarosite suggests oxidation of sulfides or short‐lived and/or local aqueous acidic conditions, which could have produced ferric amorphous sulfates in addition to the jarosite. Additional S‐bearing, X‐ray amorphous materials are possible components of all drill samples because many S‐bearing phases evolve SO_2_ at temperatures >500°C (McAdam et al., 2014). Probable S‐bearing phases include adsorbed S species and crystalline S‐bearing phases below the CheMin detection limit (Morris et al., 2016; Rampe et al., 2017). The variability of amorphous Mg‐ and Fe‐sulfates in Marimba and Quela suggest changes in acidity and evaporation at these two sites. Mg‐sulfate in Marimba suggests a stronger evaporative episode to concentrate Mg^2+^ in solution. The overall increase in amorphous sulfate abundance observed in Quela, specifically higher abundances of Fe‐sulfate (both jarosite and amorphous Fe‐sulfate), suggests that rocks at Quela experienced prolonged exposure to acidic fluids compared to Marimba.

### 4.4 Mineralogical Variability Within Gale Crater Mudstones

**Figure 10:**
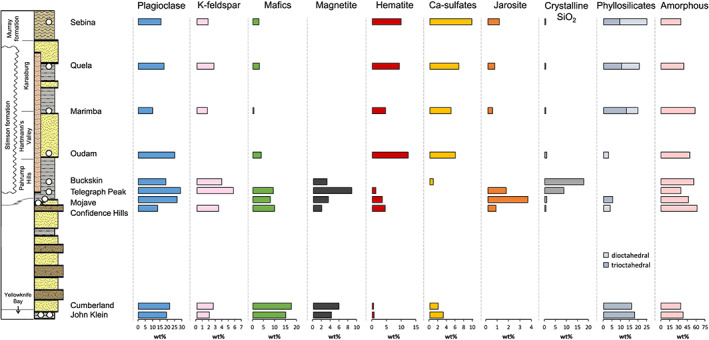
Crystalline, phyllosilicate, and amorphous material distributions for drilled samples analyzed by CheMin in Gale crater. Phase distributions shown are the maximum crystalline abundances (minimum amorphous abundance; Table S3) calculated for each sample. Data for samples stratigraphically below Oudam can be found in Vaniman et al. (2014) (Cumberland and John Klein), Rampe et al. (2017) (Confidence Hills, Mojave, Telegraph Peak, and Buckskin), and Morris et al. (2016) (Buckskin).

The variations in mineralogy, chemistry, and sedimentology from Yellowknife Bay to the Sebina drill site in the Sutton Island member underscore the complex depositional and diagenetic history of Gale crater's fluvial‐lacustrine sediments (Figure 10).

The identification of detrital, authigenic, and diagenetic crystalline phases plays an essential role in assessing changes in source sediments and interpreting the various alteration conditions that influenced Bradbury and Murray formation mudstones. The detrital mineral assemblages observed in the John Klein and Cumberland samples from Yellowknife Bay indicate a shared basaltic source (Figure 10; Vaniman et al., 2014). The lowermost Murray drill samples, Confidence Hills and Mojave2, also have similar detrital mineralogy; however, different smectite species may imply subtle variations in parent lithologies or exposure to different alteration conditions (Rampe et al., 2017; Yen et al., 2017). Abundant crystalline silica minerals (i.e., cristobalite and tridymite) were identified in Telegraph Peak and Buckskin, suggesting a change from basaltic to a more silicic source sediment supplying the lacustrine system (Morris et al., 2016; Rampe et al., 2017) or a high‐temperature, hydrothermal event (Yen et al., 2018). Lastly, the mineral assemblages in the Marimba, Quela, and Sebina mudstones, with pyroxene and trioctahedral smectites, suggest basaltic detritus (see section 4.2), and the presence of dioctahedral smectites indicates an open‐system aqueous environment (see section 4.2).

The clay mineralogy of mudstones in Gale crater serve as key indicators of variability in depositional environments. In conjunction with the detrital mineralogy, lake water conditions and episodes of fluid migration influenced the composition and structure of observed smectites from Yellowknife Bay to Sutton Island (Figure 10). In Yellowknife Bay, an isochemical, closed‐system environment resulted in the formation of Fe‐rich trioctahedral smectites (Bristow et al., 2015; McLennan et al., 2014). High abundances of clay minerals were observed in the Marimba, Quela, and Sebina mudstones, but, unlike Yellowknife Bay, the presence of both dioctahedral and Mg‐rich trioctahedral smectites suggests an open‐system lacustrine setting that was subject to evaporative concentration and episodes of wetting and drying (Bristow et al., 2018; Mangold et al., 2018; Rapin et al., 2019). Minor phyllosilicate abundances in Confidence Hills and Mojave2, coupled with the absence of smectites in Telegraph Peak and Buckskin, suggest differences in source sediments, depositional environment, and/or diagenetic conditions from those inferred for Yellowknife Bay, Karasburg, and Sutton Island mudstones. Olivine‐poor basaltic detritus could account for the low smectite abundance in Confidence Hills and Mojave; or lake water conditions may have been suboptimal for smectite formation (i.e., low salinity; Bristow & Milliken, 2011). If such lake conditions persisted during the deposition of Telegraph Peak and Buckskin, then smectite formation would not be expected, especially if the sediment source shifted to include more felsic rocks (i.e., less detrital olivine and pyroxene). Furthermore, a shift from Fe^3+^‐rich dioctahedral smectite (i.e., nontronite) in Confidence Hills to Mg‐rich trioctahedral smectite (i.e., saponite) in Mojave suggests an abrupt change in detrital source affecting the olivine and pyroxene ratios, lake water salinity or composition (e.g., Bristow & Milliken, 2011), if the clay minerals were detrital, a shift in source region, and/or different diagenetic processes. Given the short stratigraphic distance between samples (~1.2 m; Rampe et al., 2017) and evidence of acidic alteration, diagenetic change is the preferred explanation.

Morphological evidence for diagenesis is present at all drill locations analyzed by *Curiosity*, and this process is one of the most significant that resulted in the observed mineralogy. Nodules, raised ridges, and Ca‐sulfate veins represent at least two diagenetic fluid influxes in Yellowknife Bay (Stack et al., 2019). Fluids associated with the early diagenetic event were likely neutral to alkaline in pH, as evidenced by the proposed Mg‐hydroxy interlayers in the Cumberland phyllosilicate that preferentially form in more alkaline fluids (Bristow et al., 2015; Vaniman et al., 2014). Alternatively, the intercalated smectite could have formed from the transformation of saponite at elevated temperatures (Kristmannsdóttir, 1979; Pevear et al., 1982; Vaniman et al., 2014). Diagenesis in the Pahrump Hills member is supported by concretions present in the lower strata and by mineralogical evidence of multiple influxes of mildly to moderately acidic fluids (~2–6 pH) (Rampe et al., 2017). Jarosite was observed in all Pahrump Hills drill samples, excluding Buckskin, and is attributed to a late‐stage, acidic alteration event (Martin et al., 2017; Rampe et al., 2017). Concretions are observed throughout the Hartmann's Valley, Karasburg, and Sutton Island members (Sun et al., 2019). High abundances of Ca‐sulfate and the lack of associated syndepositional sedimentary features imply that the majority of these phases were precipitated from diagenetic fluids. These matrix Ca‐sulfates are proposed to have formed during an early diagenetic event associated with increased salinity of lake waters due to episodic evaporation and/or late‐stage influx of sulfate‐rich groundwaters (Figure 8). Hematite formation likely accompanied the Ca‐sulfate‐producing diagenetic events, excluding Oudam where evidence of hydrothermal hematite and anhydrite formation is proposed. In addition to matrix Ca‐sulfates and hematite in Karasburg and Sutton Island rocks, minor jarosite is observed and interpreted as a late‐stage diagenetic phase resulting from short‐lived and/or localized acidic fluid influx and/or oxidation of minor detrital sulfides.

Overall, the mineralogy of mudstones and sandstones illustrates the varied and complex history of aqueous alteration in Gale crater sediments. Basaltic materials were the primary source of sediments supplying this ancient lake, with possible exception of Telegraph Peak and Buckskin. Depositional conditions (e.g., open/closed environments and salinity) affected the abundance and chemistry of observed phyllosilicates. Diagenetic fluid conditions strongly influenced the formation and of both crystalline and X‐ray amorphous materials. From Yellowknife Bay to Sutton Island, the transition from magnetite to hematite and increase in matrix‐associated sulfates correlates to observations of enhanced, oxic diagenetic fluid activity.

## 5 Conclusions

Dynamic depositional environments and diverse diagenetic processes are captured in the mineralogy, geochemistry, and sedimentology of Gale crater mudstones and sandstones. Mineralogical variations observed in drilled rocks from Yellowknife Bay to Sutton Island were strongly influenced by depositional and diagenetic fluid compositions. Deposition of basaltic detritus in a lacustrine environment consistently leads to alteration of mafic phases to smectite, but mineral diversity is primarily controlled by diagenetic fluid interactions. Within the ~200 m of stratigraphic section summarized here, the mineral assemblages identified in CheMin XRD analyses indicate at least five distinct diagenetic fluid events:

1. Alkaline fluids in Yellowknife Bay—evidenced by Mg‐rich concretions, and Mg‐hydroxy interlayers at Cumberland; fluids may have been Mg‐rich and/or elevated in temperature (Grotzinger et al., 2015; Vaniman et al., 2014).
2. Acidic fluids in Pahrump Hills—supported by abundant jarosite at Mojave2 dated at 2.1 Ga, a decrease in mafic phases, partial oxidation of Fe‐oxides, and mobility of Zn, Ni, Mn, and Ti as evidenced from abundant Mg, Ni, S‐rich concretions (Martin et al., 2017; Rampe et al., 2017).
3. Hydrothermal fluids at Oudam—elevated temperatures supported by gray hematite, opal‐CT, and potential Fe‐pyrophyllite.
4. Extensive sulfur‐rich, oxic fluids in Hartmann's Valley, Karasburg, and Sutton Island members—evidenced by high abundance of matrix‐associated hematite and Ca‐sulfates precipitated early during lake shallowing/evaporation and/or late resulting from groundwater infiltration.
5. Ubiquitous precipitation of Ca‐sulfates in fractures—observed as veins, commonly crosscutting earlier diagenetic features (i.e., concretions).

These five events do not encompass the complete diagenetic history experienced by Gale sediments as widespread evidence of diagenetic processes has been observed throughout *Curiosity*'s traverse (e.g., Kah et al., 2015, 2018; Kronyak et al., 2019; Nachon et al., 2017). Of the distinct diagenetic events described above, at least two illustrate that sedimentary transitions play a significant role in the path of diagenetic fluids and the varied intensity of alteration surrounding sediments. For example, the Mojave2 mudstone is in stratigraphic proximity to a contact with interbedded sandstone; the Oudam sandstone was drilled just above the Pahrump Hills/Hartmann's Valley contact and is within ~0.5 m of the Murray mudstone/Stimson sandstone contact (Figure 1; S. G. Banham, personal communication, 2019). The latter example may link the fluid event associated with the formation Stimson alteration halos to the same diagenetic event that influenced Oudam (Figure 7); this event may have also played a role in the assemblages observed at Buckskin (~0.1 m from the Stimson contact; S. G. Banham, personal communication, 2019; Yen et al., 2017; Yen et al., 2018). Future drill campaigns targeting rocks near unconformities and/or lithology transitions can test the hypothesis that contacts play a significant role in diagenetic fluid flow and increased abundances of secondary/alteration minerals. These physical controls and the mineralogy and geochemistry of associated sediments are important factors when considering whether the drilled rock is truly representative of the bulk lithology or whether the mineralogy reflects increased alteration. Both are important considerations when interpreting the geologic history of martian sediments.

## Supporting information

Supporting Information S1

Table S1

Table S2

Table S3

Table S4

## Data Availability

All data used in this manuscript are available at the NASA Planetary Data System Geosciences Node. CheMin diffraction patterns and information regarding the data products are located online (at https://pds-geosciences.wustl.edu/missions/msl/chemin.htm). SAM EGA data and data product descriptions are located online (at https://pds-geosciences.wustl.edu/missions/msl/sam.htm). Additionally, CheMin diffraction patterns, SAM H2O and SO2 EGA data, Tables 1, 2, and S1–S3, are publicly available (at https://doi.org/10.17632/b8fr3c64pp.1). As a resource to the reader, CheMin diffraction patterns, structure files, and analysis results are also located on the Open Data Repository (at https://odr.io/CheMin).
