# Supplementary material for: Evidence for Multiple Diagenetic Episodes in Ancient Fluvial‐Lacustrine Sedimentary Rocks in Gale Crater, Mars
Source: J Geophys Res Planets. 2020 Aug 13;125(8):e2019JE006295. doi: 10.1029/2019JE006295 (PMC7507756; doi:10.1029/2019JE006295)
Supplement: Supplementary file 1 — Supporting Information S1 [file JGRE-125-e2019JE006295-s001.docx]

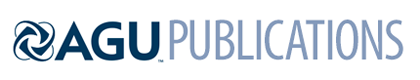


*Journal of Geophysical Research - Planets*

Supporting Information for

**Evidence for Multiple Diagenetic Episodes in Ancient Fluvial-Lacustrine Sedimentary Rocks in Gale Crater, Mars**

C. N. Achilles^1^, E. B. Rampe^2^, R. T. Downs^3^, T. F. Bristow^4^, D. W. Ming^2^, R. V. Morris^2^,

D. T. Vaniman^5^, D. F. Blake^4^, A. S. Yen^6^, A. C. McAdam^1^, B. Sutter^7^, C. M. Fedo^8^, S. Gwizd^8^,

L. M. Thompson^9^, R. Gellert^10^, S. M. Morrison^11^, A. H. Treiman^12^, J. A. Crisp^6^, T. S. J. Gabriel^13^, S. J. Chipera^14^, R. M. Hazen^11^, P. I. Craig^5^, M. T. Thorpe^2^, D. J. Des Marais^4^,

J. P. Grotzinger^15^, V. M. Tu^7^, N. Castle^5^, G. W. Downs^3^, T. S. Peretyazhko^7^, R. C. Walroth^4^,

P. Sarrazin^16^, and J. M. Morookian^6^

^1^NASA Goddard Space Flight Center, Greenbelt, MD USA, ^2^NASA Johnson Space Center, Houston, TX USA, ^3^University of Arizona, Tucson, AZ USA, ^4^NASA Ames Research Center, Moffett Field, CA USA, ^5^Planetary Science Institute, Tucson, AZ USA, ^6^Jet Propulsion Laboratory, California Institute of Technology, Pasadena, CA USA, ^7^Jacobs at NASA Johnson Space Center, Houston, TX USA, ^8^University of Tennessee-Knoxville, Knoxville, TN USA, ^9^Univeristy of New Brunswick, Fredericton, NB Canada, ^10^University of Guelph, Guelph, ON Canada

^11^Carnegie Institute for Science, Washington, D.C. USA, ^12^Lunar and Planetary Institute, Houston, TX USA

^13^Arizona State University, Tempe, AZ USA, ^14^Chesapeake Energy, Oklahoma City, OK USA, ^15^California Institute of Technology, Pasadena, CA USA, ^16^SETI Institute, Mountain View, CA USA

**Contents of this file**

Text S1

Tables S1 to S3

**Introduction**

Included in the supplemental information below are detailed methods and information regarding sample acquisition, data processing, and data analyses for the three primary instrument datasets presented in the manuscript. Tables include the bulk chemical analyses of each drill sample (Table S1), the calculated crystal chemistries for plagioclase and hematite (Table S2), and the phase distributions based on the calculated minimum amorphous abundance.

**Text S1.**

*Sample Acquisition*

The MSL drill is designed to penetrate and powder rocks for delivery to the CheMin and SAM instruments. The drill produces a ~1.6 cm diameter hole as it penetrates ~5-6 cm into the target rock. The upper ~1.5 cm of drilled rock is discarded around the drill hole and the lower ~3-4 cm is collected in the drill stem and transferred to the Collection and Handling for *In situ* Martian Rock Analysis (CHIMRA) system to generate sieved fractions (Anderson et al., 2012). Portions of the <150 µm fraction (~75 mm^3^) are generated for delivery to CheMin and SAM.

*APXS Geochemical Analyses*

APXS determines the chemical compositions of rocks and soils by X-ray spectroscopy. A ^244^Cm radioactive source irradiates a target and a silicon diode detector measures the X-rays produced by fluorescence and particle-induced emission. Within a 1.5-2 cm diameter field of view, the MSL APXS instrument can detect and quantify the elements Na to Br, with characteristic X-ray energies between ~0.7 and ~25 keV (Gellert and Clark, 2015; Thompson et al., 2016). For each selected drill target, APXS measurements of the pre-drilled rock, drill tailings, and the material not delivered to the CheMin or SAM instrument were acquired. Material remaining in CHIMRA following delivery to CheMin and SAM is dumped into two piles designated pre-sieve and post-sieve. Pre-sieve material was not processed through a 150 µm sieve and is the bulk powder acquired through the drilling process. Post-sieve material is the finest sample fraction (<150 µm) and most closely represents the bulk chemistry of material delivered to CheMin and SAM. APXS analyses of the Oudam, Marimba, Quela, and Sebina drill fines were acquired on sols 1368, 1426, 1466, and 1496, respectively. All analyses in Table S1 and used in the calculation of the X-ray amorphous chemistry were measurements of the drill tailings, not the post-sieve material. Scattering of the post-sieve fines by wind was observed in each instance therefore, the drill tailings analysis is considered the most representative composition of the sample analyzed by CheMin.

*CheMin X-ray Diffraction and X-ray Fluorescence Measurements*

The CheMin XRD/XRF instrument produces diffraction patterns and XRF spectra of scooped soils or drilled rock samples (Blake et al., 2012). Samples are sieved to <150 µm and a portion is delivered to one of the instrument’s 27 reusable sample cells. Cells hold the sample material between two polymer (Mylar or Kapton) windows and a piezoelectric actuator produces convective flow of the sample material, randomizing grain orientations and minimizing orientation effects. CheMin is a transmission diffractometer with a Co X-ray source (λ = 1.790276 Å) collimated to a 70 µm diameter X-ray beam. Two-dimensional (2D) XRD images are collected by an X-ray sensitive charge-coupled device (CCD) over 10 to 30 hours of analysis. Diffracted CoKα X-ray photons are summed to yield a 2D energy-discriminated CoKα diffraction pattern. All detected photons are also summed into a histogram that represents an XRF spectrum of the sample.

Oudam drill powder was delivered to a previously used but empty Mylar cell (cell 12a). This cell showed no significant residue after the previous drill sample was emptied, as confirmed by empty-cell analysis on sol 812. Thirty hours of data were collected over four sols (1363, 1366, 1370, and 1399). The Marimba drill sample was delivered to pristine Mylar cell 8a and 30 hours of data were acquired over four sols (1426, 1432, 1434, and 1437). Quela was analyzed in pristine Kapton cell 5a for 30 hours on sols 1472, 1476, 1479, 1481. The Sebina drill sample was delivered to the pristine Kapton cell 4b and 25.5 hours of data were collected over four sols (1499, 1502, 1504, and 1508).

The 2D diffraction images are converted to 1D diffraction patterns using a modification of the open-source GSE_ADA software (Dera et al., 2013). Initial pattern calibration is made with reference to a beryl-quartz standard, contained in one of the CheMin sample cells. Individual sample cells vary up to +/- 120 µm from the ideal diffracting position due to tolerance variations in the machining of the sample cell assemblies. An internal calibration method, based on the refined unit-cell parameters of plagioclase (see Morrison et al., 2018a for details), allows for the calculation of this offset value. Offset magnitudes are not large enough to inhibit phase identification, but the offsets do affect refined unit-cell parameters and the mineral chemistries calculated from the unit-cell values. The calculated cell offsets for Oudam, Marimba, Quela, and Sebina are -52, -113, -47, and -112 µm, respectively. Calibrated diffraction patterns reflecting the sum of all analyses are available at: Oudam - https://pds-geosciences.wustl.edu/msl/msl-m-chemin-4-rdr-v1/mslcmn_1xxx/data/rdr4/cmb_518482923rda13630542280ch00113p1.csv, Marimba - https://pds-geosciences.wustl.edu/msl/msl-m-chemin-4-rdr-v1/mslcmn_1xxx/data/

rdr4/cmb_524082694rda14260561236ch00113p2.csv, Quela - https://pds-geosciences.wustl.edu/

msl/msl-m-chemin-4-rdr-v1/mslcmn_1xxx/data/rdr4/cmb_528182076rda14720580642

ch00113p2.csv, and Sebina - https://pds-geosciences.wustl.edu/msl/msl-m-chemin-4-rdr-v1/

mslcmn_1xxx/data/rdr4/cmb_530590971rda14990582136ch00113p2.csv. Calibrated diffraction patterns reflecting the first analysis nights used to determine the distribution of Ca-sulfates prior to dehydration are located at: Marimba - https://pds-geosciences.wustl.edu/msl/msl-m-chemin-4-rdr-v1/mslcmn_1xxx/data/rdr4/cmb_524082694rda14260561236ch00113p1.csv, Quela - https://pds-geosciences.wustl.edu/msl/msl-m-chemin-4-rdr-v1/mslcmn_1xxx/data/rdr4/

cmb_528182076rda14720580642ch00113p1.csv, and Sebina - https://pds-geosciences.wustl.edu/

msl/msl-m-chemin-4-rdr-v1/mslcmn_1xxx/data/rdr4/cmb_530590971rda14990582136

ch00113p1.csv

Rietveld refinements of the calibrated patterns provide mineral abundances and unit-cell parameters of the major crystalline phases. Individual mineral chemistries are determined from refined unit-cell parameters (Morrison et al., 2018a). The abundance of clay minerals and the X-ray amorphous component was estimated from FULLPAT models of the diffraction pattern. FULLPAT uses natural and synthetic crystalline, clay, and X-ray amorphous phases as standards to estimate relative intensity contributions from crystalline, poorly-crystalline (e.g., smectites), and amorphous phases (Chipera and Bish 2013). The relative proportions of dioctahedral and trioctahedral smectites in Marimba, Quela, and Sebina were estimated with BGMN refinements. BGMN is a Rietveld refinement program that generates XRD patterns of partially disordered clay minerals and can simultaneously consider contributions from crystalline phases (Bergmann et al., 1998; Ufer et al., 2004). Structural models of dioctahedral smectite (based on montmorillonite) and a trioctahedral smectite (saponite) were refined along with crystalline phases to estimate the ratios of dioctahedral and trioctahedral smectites (see Bristow et al., 2018 for details and refinement parameters).

The chemical composition of the amorphous component was estimated from mass-balance calculations using the APXS-determined bulk composition of each sample (Table S1) and the quantity and composition of the crystalline components determined from Rietveld refinement (Morris et al., 2016, Morrison et al., 2018a). The calculated plagioclase composition (Table S2), average Gale crater orthopyroxene and augite compositions, and average sanidine composition from the first four Murray samples were used for the amorphous calculations. Ideal chemistries were assumed for all other crystalline phases. A nearly end-member, natural ferripyrophyllite of hydrothermal origin was used for Oudam (Sample #6, Badaut et al., 1992). A Mg-saponite from Ballarat, CA (Source Clay, Post, 1984) was used for trioctahedral smectites and dioctahedral smectites were represented by an Fe-rich phase with a dehydroxylation temperature most closely matching that observed in SAM (Sample #12, Brigatti et al., 1983).

Mass balance calculations resulted in negative oxide abundances for Oudam and Marimba, suggesting the amorphous fraction was underestimated in FULLPAT analyses. Several factors may result in discrepancies between FULLPAT and compositional-based amorphous estimates. 1) sample heterogeneity between the sample analyzed by CheMin/SAM and the composition of the drill tailings measured by APXS, 2) large uncertainties in the FULLPAT-estimated value (1σ is ± 25% of estimated amorphous fraction) due to the fundamentals of the technique and CheMin diffraction conditions and the (limited angular range, low resolution), 3) limited availability of clay minerals (especially trioctahedral smectites) for SAM-like laboratory EGA analyses, resulting in a narrow range of clay-mineral chemistries available for mass balance calculations, and 4) the decision assign clay minerals and opal-CT as crystalline phases. When the mass balance calculations result in negative oxide values, the minimum amorphous abundance is determined by increasing the amorphous fraction until the all oxides are non-negative values (one oxide equals zero). Oudam presents an interesting case due to the detection of opal-CT. In Table 2 and Fig. 7, opal-CT was not considered a crystalline phase resulting in the depletion of CaO at the FULLPAT-estimated amorphous fraction (~43 wt%; 35.5 wt% amorphous + 7.2 wt% opal-CT) and a zero value at ~49 wt% amorphous (the calculated minimum amorphous value). If opal-CT is considered a crystalline phase, a negative composition still results, however the contribution of CaO-bearing phases is reduced and the calculated minimum is 35.7 wt% (Table S3). Because of these situations, one must carefully consider how the calculations are performed.

|  |  | Oudam |  | Marimba |  | Quela |  | Sebina |
| --- | --- | --- | --- | --- | --- | --- | --- | --- |
| sol |  | 1368 |  | 1426 |  | 1466 |  | 1533 |
| SiO_2_ |  | 51.82 |  | 45.99 |  | 44.81 |  | 43.74 |
| TiO_2_ |  | 1.05 |  | 1.07 |  | 1.05 |  | 1.01 |
| Al_2_O_3_ |  | 9.4 |  | 8.49 |  | 8.48 |  | 8.30 |
| Cr_2_O_3_ |  | 0.32 |  | 0.33 |  | 0.29 |  | 0.35 |
| FeO_T_ |  | 18.74 |  | 22.53 |  | 18.93 |  | 18.59 |
| MnO |  | 0.22 |  | 0.09 |  | 0.22 |  | 0.19 |
| MgO |  | 4.9 |  | 4.58 |  | 4.1 |  | 4.76 |
| CaO |  | 4.55 |  | 5.27 |  | 7.48 |  | 7.54 |
| Na_2_O |  | 2.59 |  | 2.11 |  | 2.19 |  | 2.26 |
| K_2_O |  | 0.87 |  | 0.83 |  | 0.77 |  | 0.72 |
| P_2_O_5_ |  | 0.52 |  | 1.05 |  | 1.1 |  | 0.67 |
| SO_3_ |  | 4.36 |  | 6.78 |  | 9.3 |  | 10.58 |
| Cl |  | 0.35 |  | 0.48 |  | 1.01 |  | 1.05 |
| Total |  | 99.69 |  | 99.60 |  | 99.73 |  | 99.76 |

^a^Oxides and Cl are reported in wt %

**Table S1.** APXS chemical compositions of drill samples^a^

|  | *Plagioclase* | | | | | | | | | | |  | |  |
| --- | --- | --- | --- | --- | --- | --- | --- | --- | --- | --- | --- | --- | --- | --- |
|  | *a* (Å) | *b* (Å) | | *c* (Å) | | α (°) | | β (°) | | γ (°) | | chemical formula | |  |
| Oudam | 8.163(5) | 12.852(7) | | 7.110(4) | | 93.52(4) | | 116.31(4) | | 90.05(5) | | (Ca_0.40(7)_Na_0.60_)(Al_1.40_Si_2.60_)O_8_ | |  |
| Marimba | 8.161(8) | 12.849(7) | | 7.111(6) | | 93.41(6) | | 116.30(4) | | 90.09(3) | | (Ca_0.39(5)_Na_0.61_)(Al_1.39_Si_2.61_)O_8_ | |  |
| Quela | 8.175(7) | 12.845(8) | | 7.118(7) | | 93.43(5) | | 116.39(6) | | 90.06(4) | | (Ca_0.39(6)_Na_0.61_)(Al_1.39_Si_2.61_)O_8_ | |  |
| Sebina | 8.174(7) | 12.857(14) | | 7.116(7) | | 93.44(5) | | 116.28(7) | | 90.10(8) | | (Ca_0.42(6)_Na_0.58_)(Al_1.42_Si_2.58_)O_8_ | |  |
|  |  |  | |  | |  | |  | |  | |  | |  |
|  | *Hematite* | | | | | | | | | | |  | |  |
|  | *a* (Å) | | *b* (Å) | | *c* (Å) | | α (°) | | β (°) | | γ (°) | |  | |
| Oudam | 5.029(1) | | 5.029(1) | | 13.738(3) | | 90 | | 90 | | 120 | |  | |
| Marimba | 5.029(1) | | 5.029(1) | | 13.742(4) | | 90 | | 90 | | 120 | |  | |
| Quela | 5.031(2) | | 5.031(2) | | 13.755(7) | | 90 | | 90 | | 120 | |  | |
| Sebina | 5.030(2) | | 5.030(2) | | 13.748(6) | | 90 | | 90 | | 120 | |  | |

**Table S2.** Unit-cell parameters and calculated chemical formula for plagioclase feldspar and hematite

|  | *Bulk Phase Distributions* | | | |  |
| --- | --- | --- | --- | --- | --- |
|  | Oudam^b^ | Oudam^c^ | Marimba | Quela | Sebina |
| Crystalline | 54 | 48 | 25 | 40 | 41 |
| Clay Minerals | 3 | 3 | 22 | 21 | 25 |
| Opal-CT | 7 | -- | 0 | 0 | 0 |
| Min. Amorphous | 36 | 49 | 53 | 39 | 34 |
| Total | 100 | 100 | 100 | 100 | 100 |

^a^Oxides and Cl are reported in wt %

^b^Opal-CT considered a crystalline phase in amorphous composition calculations

^b^Opal-CT considered an amorphous phase in amorphous composition calculations

**Table S3.** Bulk phase distributions normalized to the minimum amorphous abundance as determined from mass balance calculations^a^
